# CT vs. MRCP in choledocholithiasis jaundice

**Published:** 2015

**Authors:** I Petrescu, AM Bratu, S Petrescu, BV Popa, D Cristian, T Burcos

**Affiliations:** * “Coltea” Clinical Hospital, “Carol Davila” University of Medicine and Pharmacy, Bucharest, Romania; ** “Floreasca” Clinical Emergency Hospital, “Carol Davila” University of Medicine and Pharmacy, Bucharest

**Keywords:** choledocholithiasis, obstructive jaundice, computed tomography (CT), magnetic resonance cholangiopancreatography (MRCP)

## Abstract

**Rationale:** Obstructive jaundice can raise problems to diagnostic imaging. The radiologist must choose the most appropriate examination that delivers the most important diagnostic information because the differences between a lithiasic obstruction and a tumoral one are vital. This information helps the surgeon speed up the process of decision-making, because the treatment may be very different in relation to the nature of the obstruction.

**Objective:** This study tries to demonstrate the diagnostic accuracy of computed tomography (CT) and magnetic resonance cholangiopancreatography (MRCP) in detecting the obstacle in the common bile duct (CBD) and the possibility of establishing the lithiasic nature of the obstruction.

**Methods and Results:** A retrospective analysis was analyzed during an interval of 18 months that included jaundice patients admitted in the General Surgery Department of “Coltea” Clinical Hospital. They were examined by CT scanning and by MRCP, being suspected of choledocholithiasis. 63 patients were included in the study, 34 females and 29 males. 33 CT scans and 30 MRCP exams were performed.

**Discussion:** CT scan is useful in detecting residual or iterative choledocholithiasis in patients after cholecystectomy, contrast enhanced CT (CECT), being able to differentiate between lithiasic and non-lithiasic obstruction. MRCP delivers important anatomic details of the biliary tree; it is superior to CT in diagnosing the hepatocholedochal lithiasis; MRCP tends to replace endoscopic retrograde cholangiopancreatography (ERCP) - the diagnostic “gold standard” reducing the number of unnecessary invasive diagnostic procedures.

**Abbreviations:** CT = computed tomography, CECT = contrast enhanced computed tomography, ERCP = endoscopic retrograde cholangiopancreatography, MRCP = magnetic resonance cholangiopancreatography, CBD = common bile duct

## Introduction

Computed tomography (CT) scan and magnetic resonance cholangiopancreatography (MRCP) are the most complex diagnostic methods of common bile duct (CBD) lithiasis, allowing the surgeon to decide rapidly the most appropriate for each individual [**1**,**2**].

CT scan can bring out details about the structure of the obstacle giving an etiological diagnosis: lithiasic or non-lithiasic obstruction, being able to establish the benign or malignant nature of the latter; can also bring out information about local or regional associated [**3**].

MRCP shows the obstacle, locating its exact position, its dimensions, the length of the obstructed CBD segment, and the degree of the upstream dilation; it also does not use X-rays [**4**,**5**].

Our aim was to evaluate the advantages and disadvantages of both methods in their ability to establish the nature of the obstacle, thus providing a helping hand in choosing each method exactly when needed, so that the diagnostic is found out sooner and the therapy to act quicker, and knowingly. 

## Methods

Our study is retrospective and it included patients from 18 months between July 2013 and December 2014. The group studied was made up of 63 patients - 34 females (53.97%) and 29 males (46.03%), admitted in the Department of General Surgery of “Coltea” Clinical Hospital for biliary disease, with scleral and tegumentary jaundice, high bilirubin levels; patients may have had prior cholecystectomy.

Patients, in whom CBD lithiasis was accidentally diagnosed, while looking for another disease, were excluded from the study.

Patients were examined by CT and MRCP. There were 33 CT scans and 30 MRCP's. CT scans were performed in 16 females and 17 males, and MRCP was performed in 18 women and 12 men. 

CT scans were made on a Siemens Somatom Emotion Duo by scanning the abdomen from above the diaphragm, downward to the level of iliac crests, by using 5 mm contiguous slices. First, a native scan was acquired, and then contrast media was used intravenously during arterial, venous and parenchymatous intervals. 

MRCP was performed on a Siemens Simphony, 1,5T, by using a MRCP technique with multiplanar reconstructions. The data was introduced in a database and statistically analyzed. 

## Results

In the CT scanned group (33 patients), 8 patients (24.24%) had previous cholecystectomy. 

Usually, a intralumenal obstruction of CBD looks like a filling defect inside the duct [**2**,**3**] or like a hyper-attenuating image on a CT scan.

The most important diagnostic conclusion: presence of calculi is shown in **Table 1**.

**Table 1:** Patients’ distribution according to the presence of calculi

	Calculi present	
	YES	NO
CT	14	19
MRCP	30	0
TOTAL	34	19

CT scan detected only “sludge” in 5 cases, but not seen as microcalculi. 

A very important aspect of choledocholithiasis is the dimensions of calculi. Because of the effects of lithiasis upon the upstream biliary tree, calculi were sorted in three groups as it follows less than 3mm, between 3-10mm, and above 10mm. This classification is presented in the table below:

**Table 2:** Patients’ distribution according to the calculi size

		Calculus size	
	<3mm	3-10mm	>10mm
CT	0	8	6
MRCP	2	28	0
TOTAL	2	36	6

In our study, we considered it very important to establish the number of calculi in the CBD, as accurately as possible, because the therapeutic methods, especially the endoscopic retrograde cholangiopancreatography (ERCP), which extracts them, needs to know exactly their number prior to the intervention. For a better analysis, the study group was divided in patients with unique calculus and patients with multiple calculi. Our sorting is presented in **Table 3**. 

**Table 3:** Patients’ distribution according to the number of calculi

	No. of calculi	
	One	Multiple
CT	8	6
MRCP	20	10
TOTAL	28	16

The exact location of the calculi in CBD is important due to its side effects on the upstream biliary tree, which may lead to a degree of liver impairment. The patients were also sorted according to the location of the calculi in the CBD, as shown in **Table 4**.

**Table 4:** Patients’ distribution according to the location of calculi

		Location of calculi	
	Papilla	Distal choledoc	Rest of choledoc
CT	2	8	4
MRCP	5	4	21
TOTAL	7	12	25

The most common consequence of choledocholithiasis is the dilation of biliary tree, upstream the obstacle. The upper limit of the choledochal caliber in non-cholecystectomy patients is of 6-8mm and in patients after cholecystectomy is of 10mm. Patients were also sorted according to the presence of dilation of the biliary tree proximal to the obstruction, as shown in **Table 5**. 

**Table 5:** Patients’ distribution according to the dilation of bile ducts

	Proximal dilation	
	YES	NO
CT	10	4
MRCP	21	9
TOTAL	31	13

## Discussion

In our study, the CT scan highlighted a number of 14 patients with calculi representing 42.42%; showed “sludge” in 5 subjects, which meant 15.16%, and did not reveal any form of lithiasis in 14 patients, meaning in 42.42%.

Much rarer CBD tumors were demonstrated in 2 patients out of 33 examined (6.06%) by CT. The data in our study showed that MRCP effectiveness was of 100%; in all 30 patients examined, we were able to demonstrate choledocholithiasis without having any details about its structure.

When CT scans depicts an obstacle in the CBD, it is of utmost importance to differentiate between calculi and tumor. This aspect of diagnosis is better depicted by contrast enhanced CT (CECT); as it was shown by Chung YE, the CBD cholangiocarcinoma can look as a filling ductal defect with an upstream dilation [**6**,**7**].

If the obstacle is of tumor nature, it will present an irregular contour; it can be accompanied by an adjacent tumoral mass outside the duct (e.g. pancreatic head tumor); the most important feature is its behavior in contrast enhanced scans: it will load with the contrast becoming hyper-attenuating [**8**,**6**]. We could not differentiate between calculi and a tumor in MRCP because there was a flow-based method that did not use contrast enhancement. This information can be obtained by MRI of the abdomen, that is not included in our study.

If the obstacle is a calculus: it can present a regular or irregular contour, generally it is not accompanied by an extralumenal tumoral mass. The characteristics of a filling defect of a lithiasic nature will also be different related to the composition of the stone.

Thus, if there is a cholesterol dominated structure, the stone will have a similar density as the bile, not being able to differentiate them apart even if the duct is dilated. It can have a mixed structure, with a slight increase in calcium, the stone will mimic the density of adjacent tissues, being very difficult to distinguish them especially when the calculus is situated distally, surrounded by pancreatic tissue. High calcium stones are the easiest to identify in non-enhanced CT scans; they appear as hyper-attenuating CBD filling defects. Lee JK stressed the low sensitivity of CT scans in demonstrating cholesterol biliary calculi [**9**,**10**].

All the calculi independent of their composition will behave similarly in CECT scans. They will never enhance, they will show the same attenuation values during the entire examination, thus assuring the perfect differential diagnostics with tumoral obstruction of CBD [**3**,**11**]. MRCP demonstrates calculi, with no contrast used, as the same filling defects, hypointense to the surrounding bile. 

Once we demonstrated the calculi, we were able to appreciate the size of the stones. In CT scans, the biggest was of 22mm, and the smallest was of 4mm; we were never able to show calculi smaller than 3mm. The most numerous were the stones between 10-13mm (6 patients out of 14-42,86%).

CT scans were performed with 5mm slices, thus being unable to identify very small biliary stones (of less than 3mm). In spite of multiplanar reconstructions, we could not find small calculi, which were supported by the medical literature data; Tsen CW stressed that calculi size affects the accuracy of CT in choledocholithiasis detection [**12**].

IN MRCP, the stone size was diverse: they were all less than 1cm wide: the smallest were of 2mm (2 patients out of 30- 6.67%), the biggest was of 9 mm (one patient out of 30 -3.33%); the majority varying between 3 and 8 mm (27 patients out of 30 - 90%).

MRCP has an overall accuracy far superior to CT scans in depicting calculi in CBD, as Norero emphasized too [**4**]. The effectiveness of MRCP is the greatest especially in very small calculi compared to CT.

Regarding the number of stones, CT can show that too. We demonstrated one, two or more calculi.

CT scans in our study showed that: in 14 patients, the most numerous subjects had one single calculus, in 8 (57.14%), but a significant number is represented by multiple calculi patients with 6 subjects out of 14 (42.86%). By comparison, ERCP demonstrated multiple stones in much more patients proving the low sensitivity of CT in this aspect. 

Like CT, MRCP can depict the number of calculi after demonstrating them first.

Our patients showed the following: one calculus - 20 patients out of 30 (66.67%); two stones - 7 patients out of 30 (23.33%); multiple calculi - 3 subjects out of 30 (10%).

**Fig. 1:**
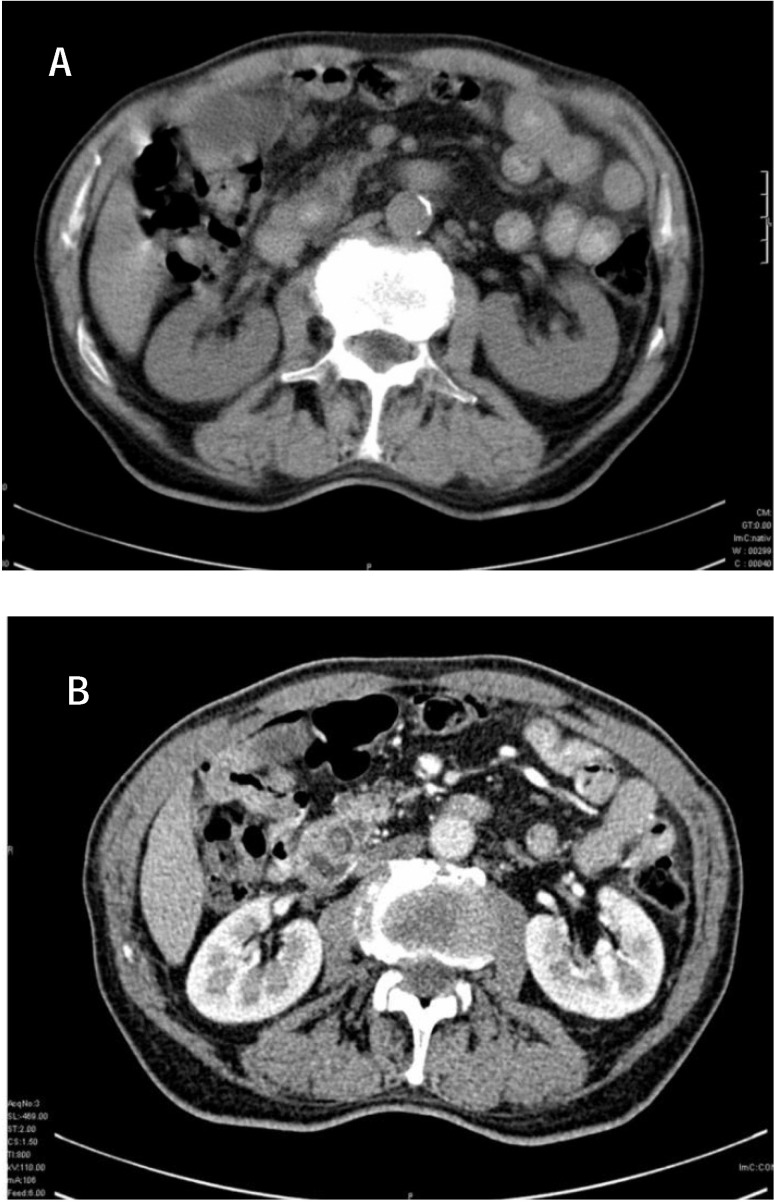
CT non-contrast (a) and contrast enhanced (b). Calculus with CBD dilation

**Fig. 2:**
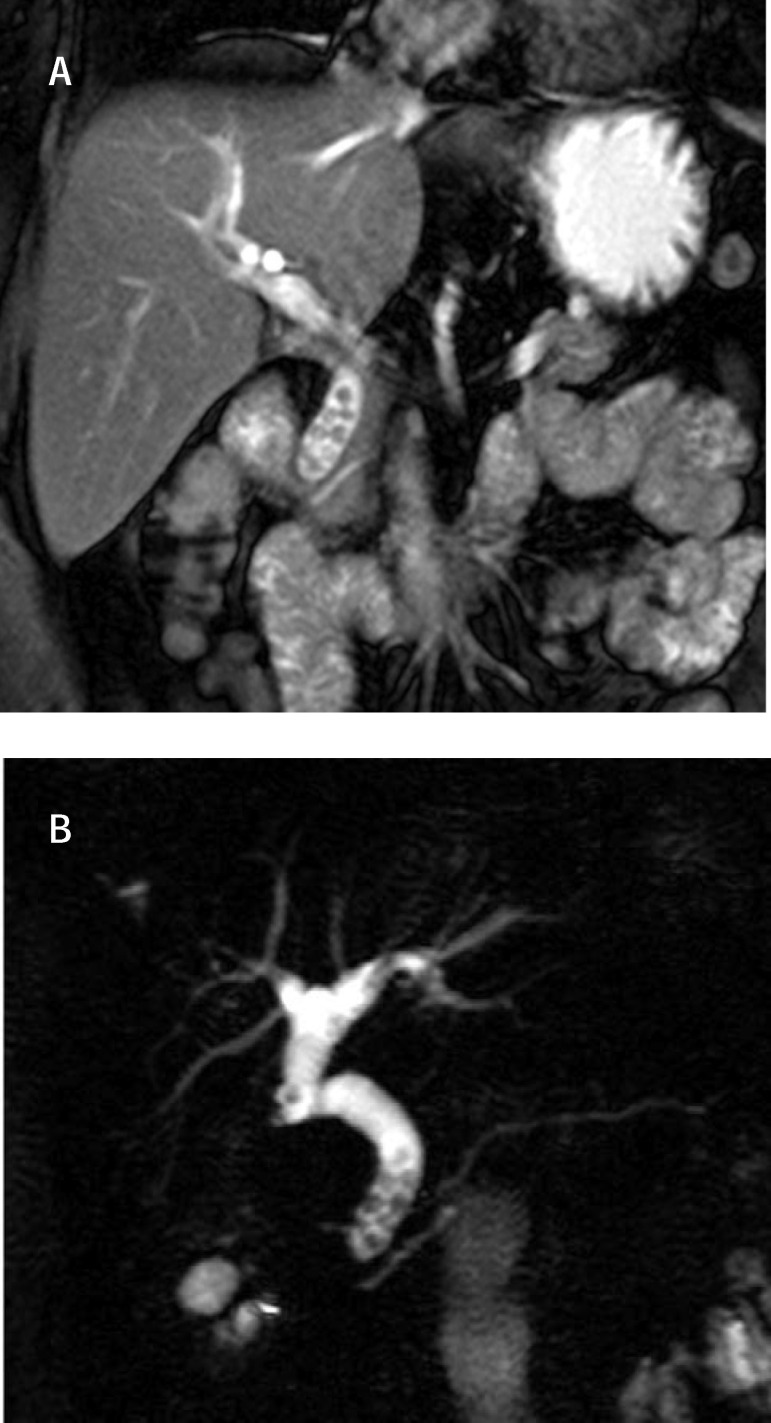
MRCP Multiple stones with dilation

After showing the calculi, depicting their size and their numbers, the next important information is their exact location. Our data showed: on CT, out of 14 patients, the location was the following: choledochal unspecific location - 4 patients (28.57%); distal choledochal location - 3 patients (21.43%); intracephalic pancreatic CBD: 5 patients (35.71%); papillary CBD: 2 patients (14.29%).

In MRCP, the location was as it follows: choledochal unspecific location - 21 patients out of 30 (70%); distal choledochal location - 2 patients out of 30 (6.67%); intracephalic pancreatic CBD - 2 patients out of 30 (6.67%); papillary calculi - 4 patients out of 30 (13.33%); both choledoc and papilla - 1 patient out of 30 (3.33%).

Papillary location was shown in 5 out of 30 patients - 16.67%, the numbers almost coincide with data from printed literature concerning ERCP [**1**,**13**,**14**].

**Fig. 3:**
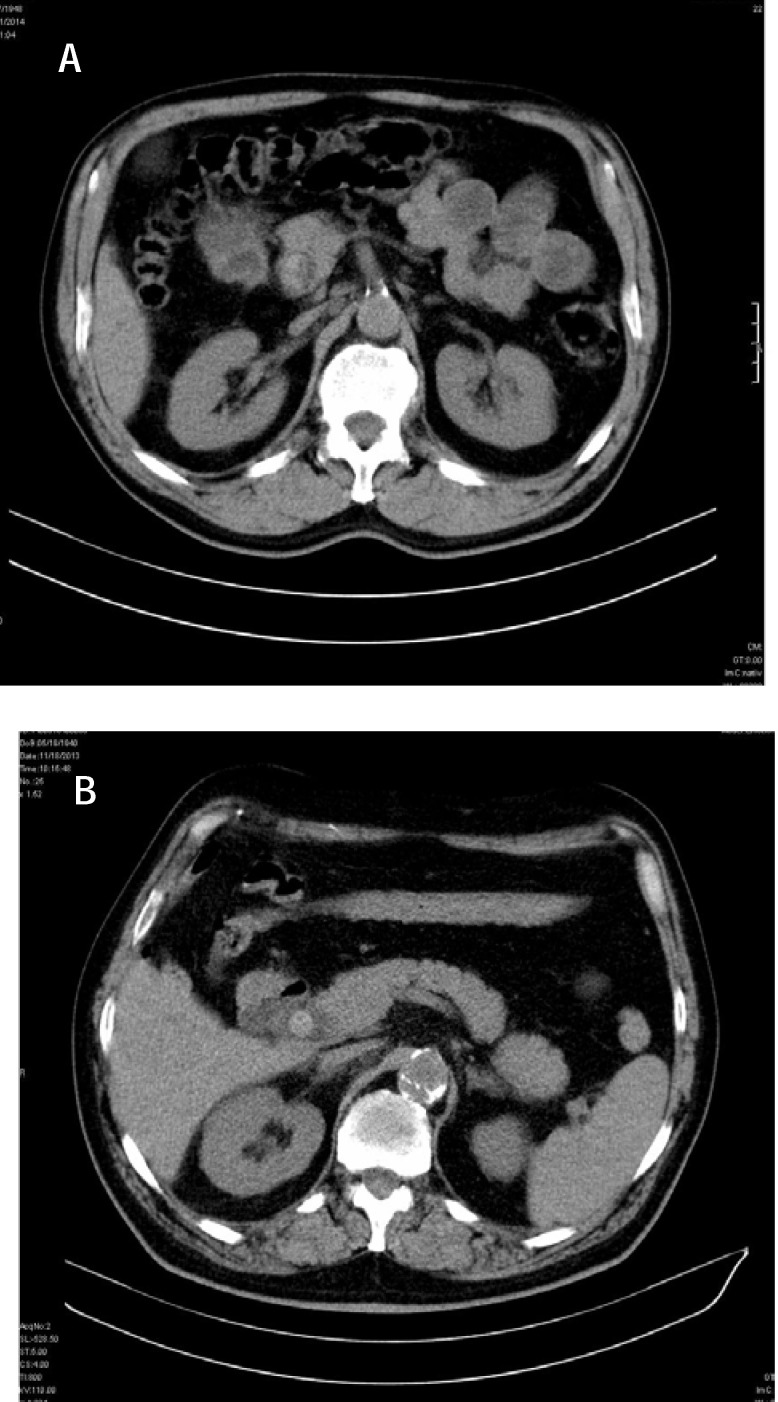
CT unenhanced: hyper attenuation calculi with dilated CBD (a) Terminal CBD; (b) prepancreatic CBD

One of the first signs that predict the biliary obstruction is the dilation of the biliary tree. This comes secondary to the presence of an obstacle and is demonstrated in more than half of the patients, in medical literature, the percentage reaching almost 75%. In our study, the ratio was 70,45%, in accordance with other literature groups of patients [**3**,**5**,**13**].

CT scans showed that 10 out of 14 patients with (71.43%) had dilated CBD of up to 24mm, the rest of 4 the patients (28.57%) had CBD of around 8-10mm.

The dilation of CBD was demonstrated in 21 patients in MRCP (21 out of 30 - 70%), the other 9 patients (30%) having between 9-10mm CBD.

**Fig. 4:**
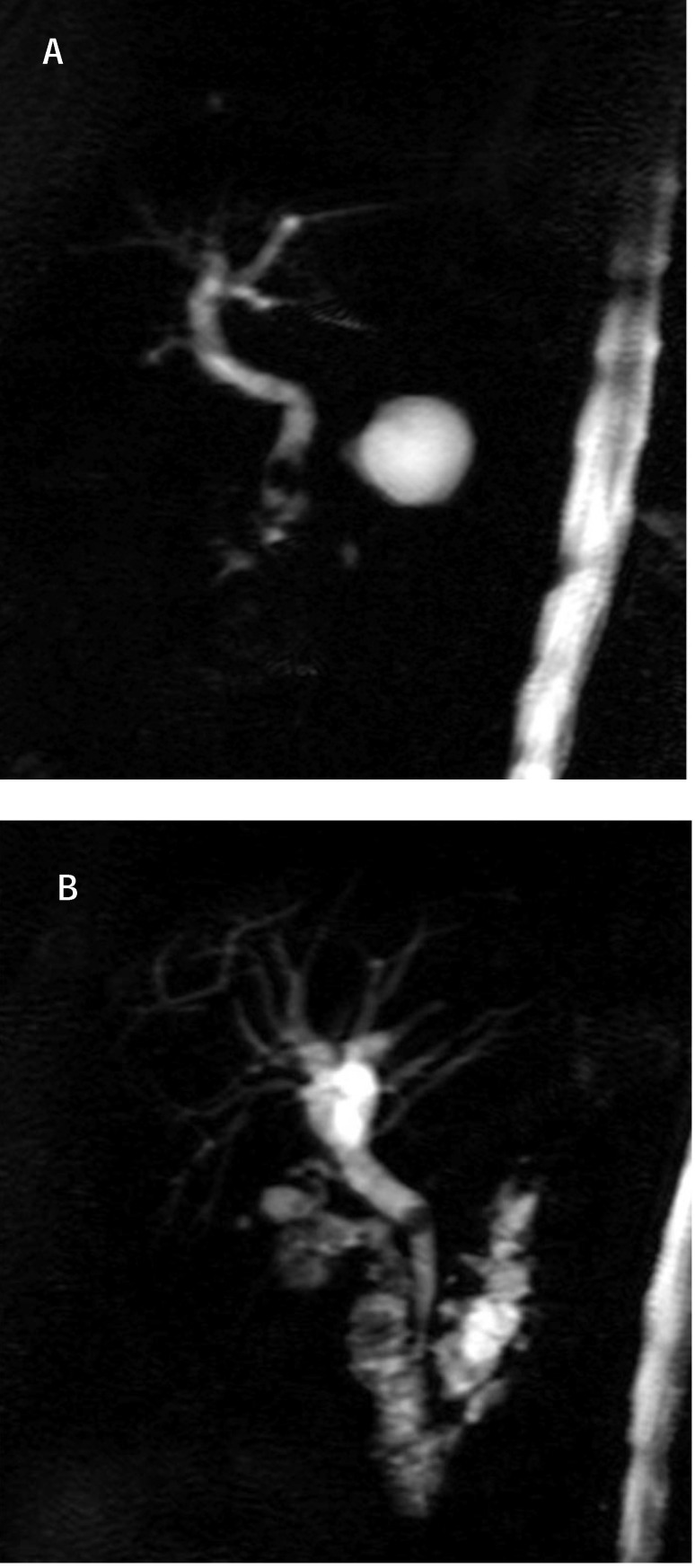
MRCP - pacient with cholecystectomy; calculus cholodoc (a) calculus with dilation of biliary tree (b)

Choledocholithiasis can start a pancreatic disease; related to the location and size of the obstacle, the patient can develop the following: an incomplete obstruction can lead to a pancreatic reaction or even a moderate acute pancreatitis due to its mere existence but also as a secondary effect in ERCP. A complete obstruction of CBD can lead to a more severe acute pancreatitis. One can encounter a reverse evolution of the disease meaning the pancreatic pathology (chronic pancreatitis, or tumoral pancreas) to start a biliary disease [**14**].

Our data showed the following: out of 33 patients scanned by CT, 11 patients (33%) were found with pancreatic disease. Three patients developed signs of mild pancreatic reaction, 3 patients showed acute pancreatitis, and other 3 patients had chronic pancreatitis. Two patients were diagnosed with pancreatic tumor.

Diagnosing one of the two main etiologies of CBD obstruction (lithiasis or tumor) can also be helped by demonstrating bilirubin levels. With a maximum normal value of 2 mg/ dL, it can vary between 2-15mg/ dL in benign obstructive jaundice, with incomplete obstruction, and can reach levels higher than 25 mg/ dL tumoral obstruction jaundice [**1**].

In spite of its effectiveness in detecting CBD lithiasis, MRCP examination cannot depict any alteration in adjacent organs, being helpful only for the biliary tree. As Gautier stressed, MRCP seems to be an efficient screening method for patients at risk for a future ERCP [**15**-**17**].

**Fig. 5:**
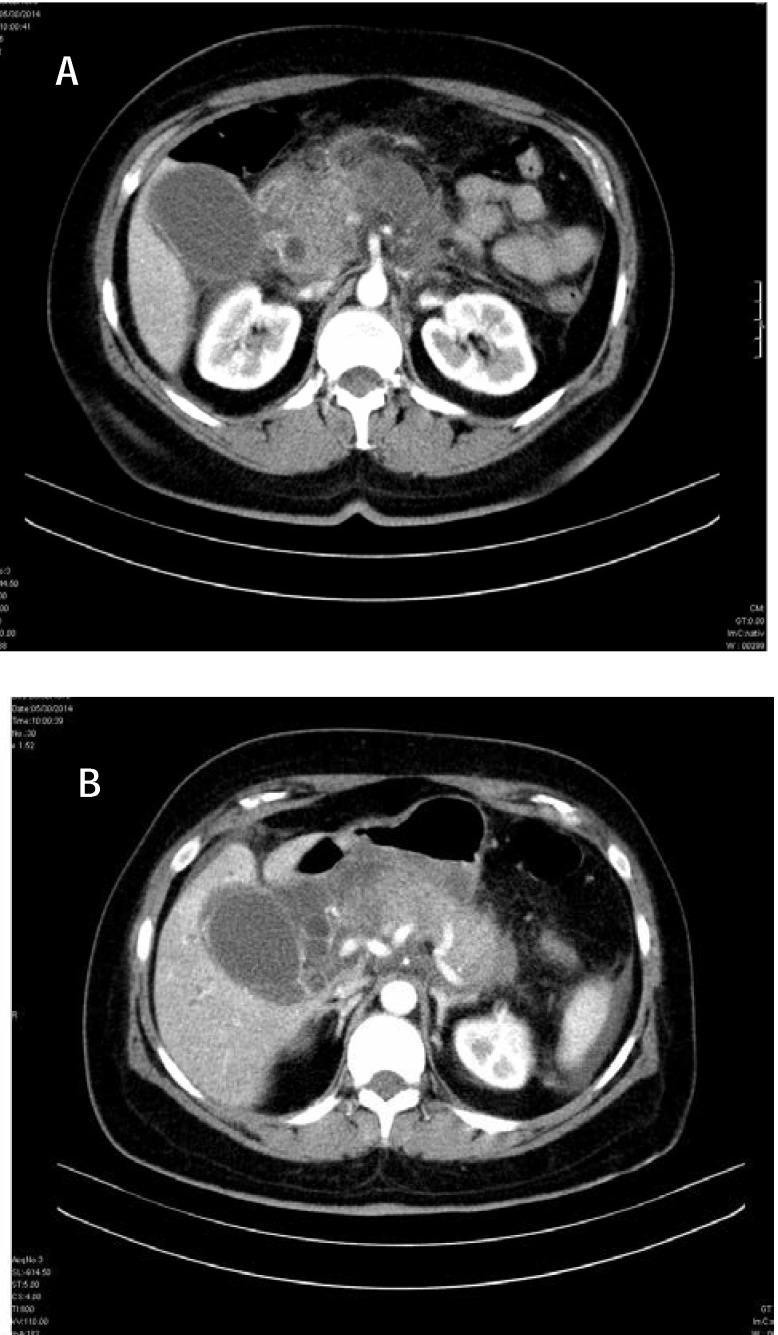
CECT Small papillary calculus, CBD dilatation, acute pancreatitis

## Conclusions

1. CT scan is important especially for patients with cholecystectomy for detecting residual or iterative calculi. 

2. CT scans do not depict biliary stones smaller than 3mm because of their limited resolution and 5 mm slices acquisition.

3. CECT can differentiate by contrast enhancement between native hyper attenuating lithiasic lesions and enhancing tumoral disease.

4. Due to the measurement of attenuation, CT scans can depict the composition and nature of biliary calculi. 

5. Because of the axial image acquisition (coronal planes are only reformatted reconstructions), CT can hardly point the location of the obstacle relating to the duodenal papilla. 

6. MRCP - a very specific investigation for biliary pathology – precisely depicts the location of the obstruction and thereafter the real distance to the papilla. Also, MRCP precisely shows the calculi number, their size (starting from 1mm); anyway MRCP cannot depict the composition of the stones. 

7. MRCP is very useful in case of biliary-digestive anastomosis because we have the possibility to acquire images in any given, desired plane: coronal, oblique sagittal, etc.

8. MRCP is a flow MRI sequence and it does not require intravenous contrast; it can fully assess biliary obstruction, but cannot demonstrate its precise nature.
